# Acute resistance exercise‐induced IGF1 expression and subsequent GLUT4 translocation

**DOI:** 10.14814/phy2.12907

**Published:** 2016-08-22

**Authors:** Kohei Kido, Satoru Ato, Takumi Yokokawa, Yuhei Makanae, Koji Sato, Satoshi Fujita

**Affiliations:** ^1^ Faculty of Sport and Health ScienceRitsumeikan UniversityKusatsuJapan; ^2^ Laboratory of Sports and Exercise MedicineGraduate School of Human and Environmental StudiesKyoto UniversityKyotoJapan; ^3^ Department of Physical EducationNational Defense AcademyYokosukaJapan; ^4^ Graduate School of Human Development and EnvironmentKobe UniversityKobeJapan

**Keywords:** Aerobic exercise, glucose transporter type 4, insulin‐like growth factor 1, resistance exercise

## Abstract

Acute aerobic exercise (AE) is a major physiological stimulus for skeletal muscle glucose uptake through activation of 5′ AMP‐activated protein kinase (AMPK). However, the regulation of glucose uptake by acute resistance exercise (RE) remains unclear. To investigate the intracellular regulation of glucose uptake after acute RE versus acute AE, male Sprague–Dawley rats were divided into three groups: RE, AE, or nonexercise control. After fasting for 12 h overnight, the right gastrocnemius muscle in the RE group was exercised at maximum isometric contraction via percutaneous electrical stimulation (3 × 10 sec, 5 sets). The AE group ran on a treadmill (25 m/min, 60 min). Muscle samples were taken 0, 1, and 3 h after completion of the exercises. AMPK, Ca^2+^/calmodulin‐dependent protein kinase II, and TBC1D1 phosphorylation were increased immediately after both forms of exercise and returned to baseline levels by 3 h. Muscle IGF1 expression was increased by RE but not AE, and maintained until 3 h after RE. Additionally, Akt and AS160 phosphorylation were sustained for 3 h after RE, whereas they returned to baseline levels by 3 h after AE. Similarly, GLUT4 translocation remained elevated 3 h after RE, although it returned to the baseline level by 3 h after AE. Overall, this study showed that AMPK/TBC1D1 and IGF1/Akt/AS160 signaling were enhanced by acute RE, and that GLUT4 translocation after acute RE was more prolonged than after acute AE. These results suggest that acute RE‐induced increases in intramuscular IGF1 expression might be a distinct regulator of GLUT4 translocation.

## Introduction

Aerobic exercise (AE) is a major physiological stimulus that induces skeletal muscle glucose uptake and improves insulin sensitivity. Acute and chronic AE affect glucose metabolism differently. Specifically, acute AE induces skeletal muscle glucose uptake incrementally and transiently (Goodyear et al. 1990). A previous study suggests that the blood glucose level is decreased approximately 30% for 4 h by acute AE, as compared with nonexercised controls (Bacchi et al. 2012). Daily blood glucose control is critical for diabetes patients to avoid diabetic complications (American Diabetes Association 2015b). Chronic AE improves insulin sensitivity, fasting blood glucose, and HbA1c levels continuously (Umpierre et al. 2011; Mann et al. 2014). The improvement of these parameters can lead to a complete reversal of type II diabetes. Thus, both acute and chronic AE are significant for improving glucose metabolism. In a recent study, chronic resistance exercise (RE), which is a skeletal muscle hypertrophy model, also improved hyperglycemia and HbA1c levels in diabetic patients (Umpierre et al. 2011). Therefore, recent exercise guidelines from the American Diabetes Association recommend RE for controlling blood glucose (American Diabetes Association 2015a). However, acute RE‐induced augmentation of skeletal muscle glucose uptake and the regulation of this process are still poorly understood.

AMP‐activated protein kinase (AMPK), which is activated by increases in the cellular AMP/ATP ratio, is a key intracellular signaling protein for glucose uptake (Musi et al. 2003; Jessen and Goodyear 2005; O'Neill 2013). The activation of AMPK is mediated by acute AE that induces glucose transporter type 4 (GLUT4) translocation to the cell membrane, resulting in glucose uptake (Musi et al. 2003; Jessen and Goodyear 2005; O'Neill et al. 2011; O'Neill 2013), but no study confirmed the signaling response after RE. Ca^2+^/calmodulin‐dependent protein kinase II (CaMKII) is another important signaling protein for glucose uptake following acute AE. Previous studies demonstrated that CaMKII activates AMPK as an upstream event in response to AE or muscle contraction (Raney and Turcotte 2008; Morales‐Alamo et al. 2013), but other studies indicated that CaMKII may also induce glucose uptake independent of AMPK signaling pathways (Raney and Turcotte 2008; Witczak et al. 2010). Although CaMKII may have important roles in skeletal muscle glucose uptake following AE, the regulation of CaMKII signaling remains unclear. Some previous studies showed that both AMPK and CaMKII were phosphorylated following acute RE, which might contribute to glucose uptake (Witczak et al. 2010; Ogasawara et al. 2014). However, no study showed the roles of AMPK and CaMKII in acute RE‐induced glucose uptake.

Phosphorylation of TBC1D1 and AS160 occurs downstream of AMPK and CaMKII (Funai and Cartee 2008; Vendelbo et al. 2014). Through the phosphorylation of TBC1D1 and AS160, AMPK and CaMKII enhance GLUT4 translocation (Chavez et al. 2008; Witczak et al. 2010). However, the relationship between AMPK/CaMKII phosphorylation and TBC1D1/AS160 phosphorylation remains unknown. Moreover, acute RE‐induced TBC1D1/AS160 responses are not fully clarified.

Insulin‐like growth factor 1 (IGF1) is increased in skeletal muscle by acute RE (Ogasawara et al. 2013a,b). In contrast, there is no evidence of acute AE inducing the expression of skeletal muscle IGF1. In a cell culture study, IGF1 phosphorylated Akt which then phosphorylated AS160 at Thr642 resulting in enhanced GLUT4 translocation and glucose uptake (Ciaraldi et al. 2002; Roach et al. 2007; Baus et al. 2008; Taylor et al. 2008; Morissette et al. 2009; Peck et al. 2009). Accordingly, IGF1 may play crucial roles as a stimulator of both AMPK‐ and CaMKII‐independent glucose uptake through the activation of Akt and AS160 signaling pathways. In general, it is poorly understood how different modes of exercise, that is, acute RE and AE, differentially regulate IGF1/Akt/AS160 signaling and subsequent GLUT4 translocation.

The purpose of this study was to identify specific cellular signal responses to acute RE, including IGF1 signaling, as compared with those to acute AE in respect with glucose metabolism. We additionally investigated the role of IGF1 on TBC1D1/AS160 phosphorylation and glucose uptake by using an in vitro model.

## Materials and Methods

### In vitro experiments

Mouse C2C12 myoblasts (American Type Culture Collection, Manassas, VA) were cultured as described previously (Yokokawa et al. 2015). Briefly, cells were grown in Dulbecco's modified Eagle's medium (DMEM; 4.5 g glucose/L, Nacalai Tesque, Kyoto, Japan) containing 10% fetal bovine serum and 1% penicillin‐streptomycin (P/S). To initiate myogenic differentiation, the culture medium was replaced by DMEM containing 2% horse serum and 1% P/S. After 4 days of differentiation, myotubes were serum‐starved overnight and then incubated for 30 min in serum‐free medium containing 5 μmol/L 5‐aminoimidazole‐4‐carboxamide ribonucleoside (AICAR; Wako, Osaka, Japan), 1 μmol/L insulin (Novolin; Novo Nordisk, Bagsvaerd, Denmark), or 200 ng/mL IGF1 (PeproTech, Rocky Hill, NJ).

### In vivo experiments

The study protocol was approved by the Ethics Committee for Animal Experiments at Ritsumeikan University, and was conducted in accordance with the Declaration of Helsinki. Forty‐two male Sprague–Dawley rats, aged 10 weeks (320–360 g), were obtained from CLEA Japan (Tokyo, Japan). The rats were divided into three groups: nonexercise control, RE, or AE. All rats were housed for 1 week in an environment maintained at 22–24°C with a 12:12‐h light–dark cycle, and were allowed food (CE2; CLEA Japan) and water ad libitum.

The time course of changes in signaling protein levels was evaluated following RE and AE initiated after a 12‐h overnight fast, as detailed below. Rats were sacrificed 0, 1, or 3 h after completion of the exercise routine. Control rats were sacrificed at the basal state. Dissected gastrocnemius muscles were frozen rapidly in liquid nitrogen and stored at −80°C until use.

### RE protocol

Under isoflurane anesthesia, hair was shaved off the right lower leg of each rat; the area was then cleaned with alcohol wipes. The rats were kept in a prone position with their right foot on the footplate and the ankle joint angle positioned at 90°. The triceps surae muscle was stimulated percutaneously with electrodes (Vitrode V, Ag/AgCl; Nihon Kohden, Tokyo, Japan) that were cut to 10 × 5 mm and connected to an electric stimulator and an isolator (SS‐104J; Nihon Kohden) (Nakazato et al. 2010). The right gastrocnemius muscle was exercised isometrically by stimulation with ten 3‐sec contractions per set for 5 sets. There was a 7‐sec interval between contractions and 3‐min rest intervals between sets. Voltage (~30 V) and stimulation frequency (100 Hz) were adjusted to produce maximal isometric tension. This exercise protocol is used widely as a RE model for animals (Ogasawara et al. 2013a,b, 2014; Tsutaki et al. 2013; Kido et al. 2015) and induces significant muscle hypertrophy (Ogasawara et al. 2013a,b).

### AE protocol

Rats in the AE group were habituated to the treadmill by running for 30 min at 15 m/min, 45 min at 20 m/min, and 60 min at 25 m/min over a week. Three to five days after the last running habituation, rats were placed on a flat treadmill and made to run for 60 min at 25 m/min (Langfort et al. 1996).

### Analyses

#### In vitro glucose uptake assay

Glucose uptake was determined by measuring the glucose concentration of the medium as described previously (Yokokawa et al. 2015). In brief, culture media were collected and the glucose concentrations assayed spectrophotometrically using a Glucose II test kit (Wako).

#### Measurements of serum IGF1, insulin, and glucose, and muscle IGF1 concentrations

Insulin‐like growth factor 1 levels in the serum and skeletal muscle were determined using the mouse/rat IGF1 Quantikine ELISA kit (R&D Systems, Minneapolis, MN). Serum insulin levels were detected using a rat insulin ELISA kit (Shibayagi, Gunma, Japan), according to the manufacturer's instructions. Serum glucose concentrations were measured by the YSI 2300 STAT Plus analyzer (Yellow Springs Instrument, Yellow Springs, OH).

#### Western blotting analyses

Western blotting analyses were performed as reported previously (Goodman et al. 2011). Briefly, stimulated C2C12 myotubes were washed once with cold phosphate‐buffered saline (PBS) and lysed in radioimmunoprecipitation assay buffer containing 10 mmol/L Tris HCl (pH 7.4), 1% NP‐40, 1% sodium deoxycholate, 0.1% sodium dodecyl sulfate (SDS), 150 mmol/L NaCl, and 5 mmol/L EDTA. The extracts were centrifuged at 13,700 ***g*** for 20 min at 4°C. Protein concentrations of the supernatants were determined using a protein assay kit (Nacalai Tesque). The lysates were mixed with 6× sample buffer containing 350 mmol/L Tris·HCl (pH 6.8), 10% SDS, 30% glycerol, 9.3% dithiothreitol, and 0.03% bromophenol blue, then boiled at 95°C for 5 min.

Gastrocnemius muscles were homogenized in buffer containing 100 mmol/L Tris·HCl (pH 7.8), 1% NP‐40, 0.1% SDS, 0.1% sodium deoxycholate, 1 mmol/L EDTA, 150 mmol/L NaCl, and protease and phosphatase inhibitor cocktail (Thermo Fisher Scientific, Waltham, MA). Homogenates were centrifuged at 13,700 ***g*** for 20 min at 4°C. The supernatant was removed, and the protein concentration determined using the Protein Assay Rapid kit (Wako).

Samples were diluted in 3× sample buffer (1.0% *β*‐mercaptoethanol, 4.0% SDS, 0.16 mol/L Tris·HCl (pH 6.8), 43% glycerol, and 0.2% bromophenol blue), and boiled at 95°C for 5 min. Using 8–12% SDS‐polyacrylamide gels, 5 *μ*g (for cell lysates) or 20 *μ*g of protein (for muscle lysates) was separated by electrophoresis and transferred to polyvinylidene difluoride membranes. After the transfer, membranes were washed in Tris‐buffered saline containing 0.1% Tween 20 (TBST). Membranes were then blocked with 5% powdered milk in TBST for 1 h at room temperature. After blocking, the membranes were washed and incubated overnight at 4°C with primary antibodies against p‐Akt (Thr308), p‐Akt (Ser473), total Akt, p‐AMPK (Thr172), total AMPK, p‐AS160 (Thr642), total AS160, p‐CaMKII (Thr286), *α*‐tubulin (Cell Signaling Technology, Danvers, MA), total CaMKII (Santa Cruz Biotechnology, Santa Cruz, CA), or p‐TBC1D1 (Ser237) (Merck Millipore, Damstadt, Germany). The membranes were then washed again in TBST and incubated for 1 h at room temperature with the appropriate secondary antibodies. Chemiluminescent reagents (Luminata Forte Western HRP Substrate; Merck Millipore) were used to facilitate the detection of protein bands. Images were scanned using a chemiluminescence detector (ImageQuant LAS 4000; GE Healthcare, Buckinghamshire, UK). Band intensities were quantified using ImageJ 1.46 software (National Institutes of Health, Bethesda, MD).

To assess the plasma membrane localization of GLUT4, plasma membranes were extracted as modified methods of previous study (Sato et al. 2009). Briefly, gastrocnemius muscles were homogenized into buffer A (20 mmol/L Tris [pH 7.4], 1 mmol/L EDTA, 0.25 mmol/L EGTA, 0.25 mol/L sucrose, 1 mmol/L DTT, 50 mmol/L NaF, 25 mmol/L sodium pyrophosphate, and 40 mmol/L *β*‐glycerophosphate). The homogenates were centrifuged at 400 ***g*** for 15 min at 4°C. The supernatant was centrifuged again at 249,138 ***g*** (Hitachi CS100GXII, Ibaraki, Japan) for 1 h at 4°C. The fractions were resuspended in buffer A and then homogenized to add equal volume buffer B containing 20 mmol/L Tris (pH 7.4), 1 mmol/L EDTA, 0.25 mmol/L EGTA, 2% Triton X‐100, 50 mmol/L NaF, 25 μmol/L sodium pyrophosphate, and 40 mmol/L *β*‐glycerophosphate. The homogenates were centrifuged at 274,052 ***g*** for 1 h at 4°C (Hitachi CS100GXII) and the supernatant was used as plasma membrane fraction. Prepared plasma membrane fraction was used for the measurement of plasma membrane GLUT4 level, which was determined by Western blotting analysis using antibodies against GLUT4 (Merck Millipore) (Sato et al. 2009).

### Statistical analysis

All results are expressed as means ± SE. A one‐way ANOVA with a least significant difference post hoc test was used to evaluate changes among multiple groups, and the unpaired Student *t‐*test was used for two‐group comparisons (Yang et al. 2012).

## Results

### In vitro experiments

#### AICAR stimulation

**Figure 1:**
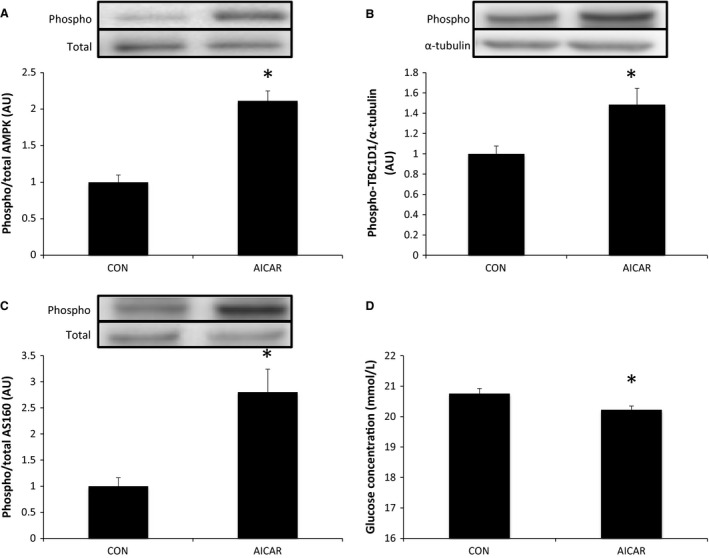
Effects of AICAR on mouse C2C12 myotubes. Phosphorylation of AMPK at Thr172 (A), TBC1D1 at Ser237 (B), and AS160 at Thr642 (C) following stimulation with AICAR. Media glucose concentration indicating glucose uptake (D) relative to control (CON) following stimulation with AICAR. Data are presented as means ± SE (*n* = 5). *P < 0.05 versus CON.

AICAR, an AMPK activator, significantly increased the phosphorylation of AMPK (Thr172), TBC1D1 (Ser237), and AS160 (Thr642). Additionally, AICAR induced significant decreases in media glucose concentrations indicating an increase in glucose uptake (Fig. 1).

#### Insulin and IGF1 stimulation

**Figure 2:**
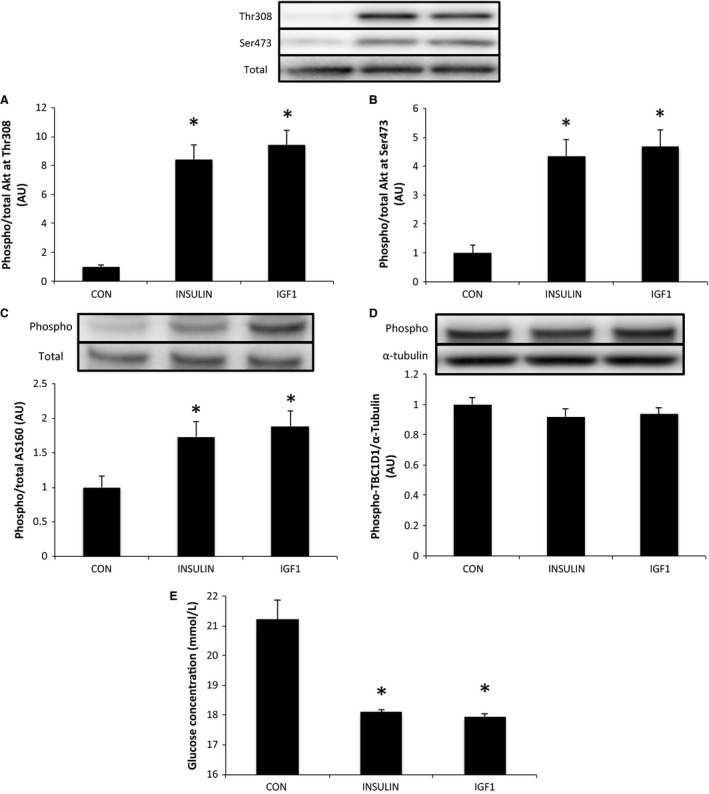
Effects of insulin and IGF1 on mouse C2C12 myotubes. Phosphorylation of Akt at Thr308 (A) and Ser473 (B), AS160 at Thr642 (C), and TBC1D1 at Ser237 (D) following stimulation by insulin or IGF1. Media glucose concentration indicating glucose uptake (E) relative to control (CON) following stimulation by insulin or IGF1. Data are expressed as means ± SE (*n* = 6). *P < 0.05 versus CON.

Akt phosphorylation at Thr308 and Ser473, and AS160 phosphorylation at Thr642, were increased significantly by stimulation with insulin and IGF1, whereas TBC1D1 phosphorylation at Ser237 was not changed. Additionally, insulin and IGF1 induced significant decreases in glucose concentrations in the media indicating an increase in glucose uptake (Fig. 2).

### In vivo experiments

#### Blood parameters

Table 1 shows the changes in serum insulin, IGF1, and glucose levels. Serum insulin was not changed by RE and AE. Serum IGF1 was decreased significantly at 3 h after AE (*P* < 0.05) but not changed by RE. In the RE group, serum glucose was increased significantly immediately after exercise (*P* < 0.05), and returned to the baseline level by 3 h after exercise. In the AE group, serum glucose was increased significantly immediately after exercise (*P* < 0.05). Glucose dropped to below the baseline level at 1 h (*P* < 0.05) and returned to the baseline level by 3 h after AE.

**Table 1:** Blood parameters

	CON	RE			AE		
		0H	1H	3H	0H	1H	3H
Insulin (pmol/l)	11.3 ± 3.0	9.7 ± 1.4	7.7 ± 3.2	12.7 ± 2.1	10.3 ± 1.8	10.9 ± 1.0	9.12 ± 0.9
IGF1 (ng/mL)	1281.4 ± 51.6	1203.9 ± 115.6	1262.3 ± 60.1	1200.0 ± 54.1	1491.9 ± 57.1	1178.1 ± 115.3	937.9 ± 72.7^a^
Glucose (mmol/L)	3.7 ± 0.3	7.2 ± 0.8^a^	9.9 ± 1.0^a^	4.0 ± 1.7	7.4 ± 0.3^a^	2.6 ± 0.1^a^	3.0 ± 0.2

Data are presented as means ± SE (*n* = 6).

CON, control; RE, resistance exercise; AE, aerobic exercise.

a *P* < 0.05 versus CON.

#### Skeletal muscle parameters

**Figure 3:**
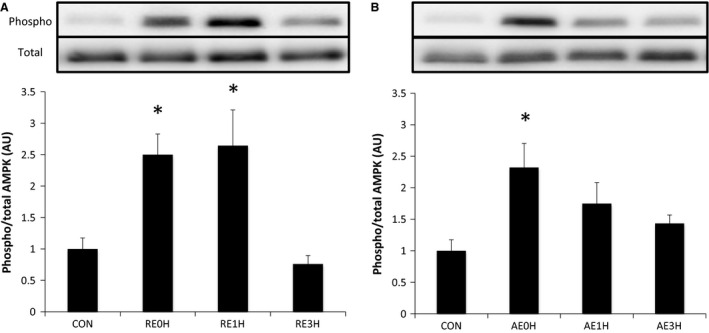
Effects of RE and AE on AMPK. Phosphorylation of AMPK at Thr172 relative to total AMPK protein content following RE (A) or AE (B). Data are expressed as means ± SE (*n* = 6). *P < 0.05 versus CON. CON, control; RE, resistance exercise; AE, aerobic exercise.

AMPK was phosphorylated significantly immediately after both RE and AE (*P* < 0.05). AMPK phosphorylation remained high at 1 h after RE (*P* < 0.05). This was in contrast to AE‐induced AMPK phosphorylation that returned to baseline by 1 h after exercise (Fig. 3).

**Figure 4:**
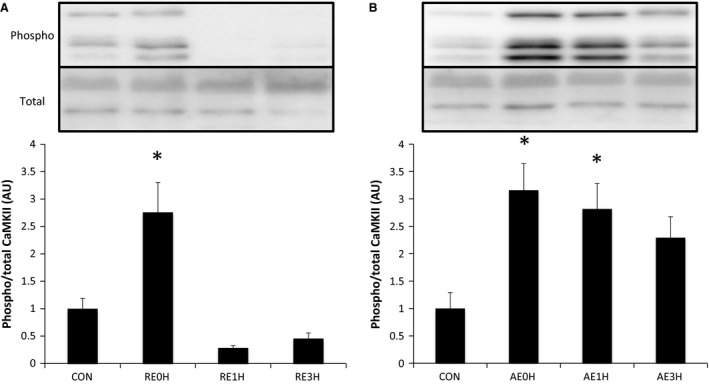
Effects of RE and AE on CaMKII. Phosphorylation of CaMKII at Thr286 relative to total CaMKII protein content following RE (A) or AE (B). Data are expressed as means ± SE (*n* = 6). *P < 0.05 versus CON. CON, control; RE, resistance exercise; AE, aerobic exercise.

CaMKII phosphorylation was increased significantly immediately after RE and returned to the baseline level by 1 h after exercise. In the AE group, CaMKII was phosphorylated significantly immediately after exercise and returned to the baseline level at 3 h after AE (Fig. 4).

**Figure 5:**
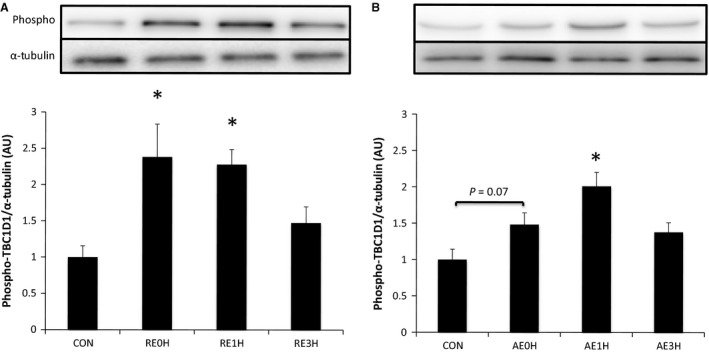
Effects of RE and AE on TBC1D1. Phosphorylation of TBC1D1 at Ser237 relative to total TBC1D1 protein content following RE (A) or AE (B). Data are expressed as means ± SE (*n* = 6). *P < 0.05 versus CON. CON, control; RE, resistance exercise; AE, aerobic exercise.

TBC1D1 was phosphorylated significantly at Ser237 immediately after RE. The phosphorylation level returned to baseline by 3 h after RE. In the AE group, TBC1D1 phosphorylation tended to increase immediately after exercise and reached significance at 1 h. Phosphorylation levels returned to baseline by 3 h after exercise (Fig. 5).

**Figure 6:**
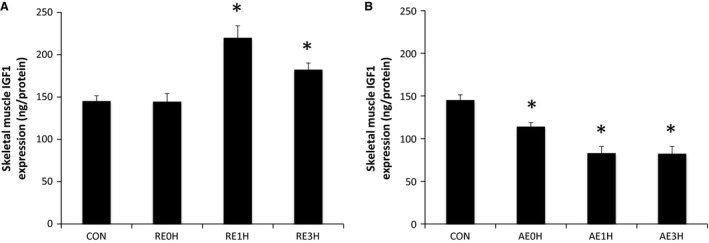
Effects of RE and AE on IGF1. The expression of skeletal muscle IGF1 relative to CON following RE (A) or AE (B). Data are expressed as means ± SE (*n* = 6). *P < 0.05 versus CON. CON, control; RE, resistance exercise; AE, aerobic exercise.

Resistance exercise induced a significant (63%) increase in IGF1 expression in skeletal muscle at 1 h after exercise. This increase was maintained until 3 h after exercise. However, AE‐induced IGF1 expression was decreased significantly at every time point (*P* < 0.05) (Fig. 6).

**Figure 7:**
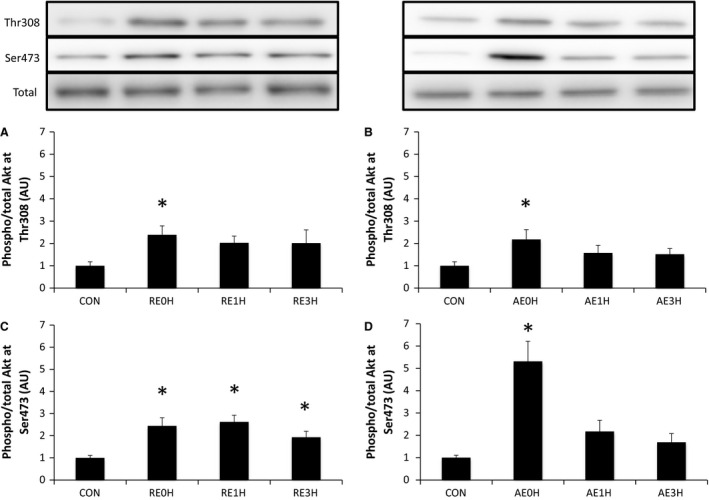
Effects of RE and AE on Akt. Phosphorylation of Akt at Thr308 and Ser473 relative to total Akt protein content following RE (A and C) or AE (B and D). Data are expressed as means ± SE (*n* = 6). *P < 0.05 versus CON. CON, control; RE, resistance exercise; AE, aerobic exercise.

Both RE and AE induced significant phosphorylation of Akt at Thr308 immediately after exercise (*P* < 0.05). p‐Akt (Thr308) levels returned to baseline by 1 h after exercise. Akt was also phosphorylated at Ser473 immediately after RE and AE (*P* < 0.05). Although AE‐induced p‐Akt (Ser473) returned to the baseline level at 1 h after exercise, this phosphorylation was maintained for 3 h after RE (*P* < 0.05) (Fig. 7).

**Figure 8:**
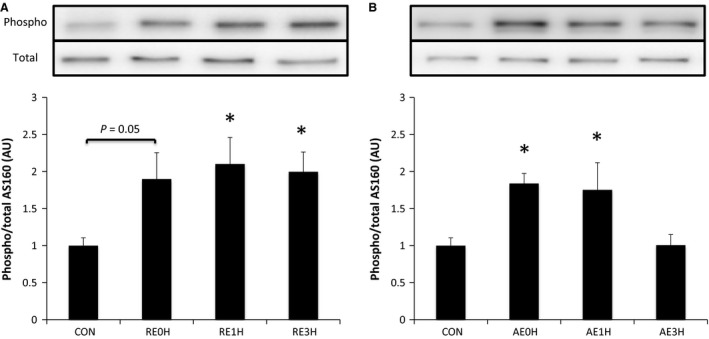
Effects of RE and AE on AS160. Phosphorylation of AS160 at Thr642 relative to total AS160 protein content following RE (A) or AE (B). Data are expressed as means ± SE (*n* = 6). *P < 0.05 versus CON. CON, control; RE, resistance exercise; AE, aerobic exercise.

Phosphorylation of AS160 at Thr642 increased significantly at 1 h after RE (*P* < 0.05). This increase was sustained for 3 h (*P* < 0.05). In contrast, AE‐induced phosphorylation of AS160 occurred immediately after exercise (*P* < 0.05) and returned to the baseline level by 3 h (Fig. 8).

**Figure 9:**
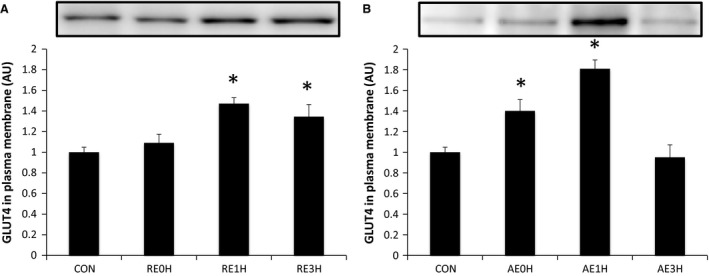
Effects of RE and AE on GLUT4. Total GLUT4 expression in plasma membrane following RE (A) or AE (B) relative to CON. Data are expressed as means ± SE (*n* = 6). *P < 0.05 versus CON. CON, control; RE, resistance exercise; AE, aerobic exercise.

The expression of GLUT4 on the plasma membrane was increased significantly 1 h after RE (*P* < 0.05). This increase was maintained until 3 h after RE (*P* < 0.05). AE induced a significant increase in plasma membrane GLUT4 immediately and 1 h after exercise (*P* < 0.05). Plasma membrane GLUT4 expression returned to the baseline level by 3 h after AE (Fig. 9).

## Discussion

In this study, we investigated intracellular signaling and GLUT4 translocation in response to an acute bout of RE or AE in rat skeletal muscle. Skeletal muscle IGF1 expression was increased after RE but not AE, and subsequent GLUT4 translocation was sustained longer after RE as compared with AE. Additionally, we used an in vitro study to show that activation of AMPK/TBC1D1 and IGF1/Akt/AS160 signaling enhanced glucose uptake independently. These data provided crucial new information to explain the regulation of glucose uptake by acute RE through specific signals, and showed differences between acute RE and AE at the molecular level.

The results of this study indicated that RE‐ and AE‐induced AMPK phosphorylation were similar. In previous studies, AMPK was also found to be phosphorylated immediately after exercise by both RE and AE, and no significant difference was observed between the responses to RE and AE (Rasmussen et al. 1998; McConell et al. 2008; Vissing et al. 2013; Ogasawara et al. 2014; Ahtiainen et al. 2015). The activation of AMPK was found previously to depend upon skeletal muscle contraction tension (Ihlemann et al. 1999). Additionally, the phosphorylation of AMPK depended upon the intensity of AE (i.e., %VO_2_max) (Chen et al. 2003; Sriwijitkamol et al. 2007). According to these previous studies, the magnitude of AMPK phosphorylation in response to RE and AE depends on exercise intensity. Therefore, further studies are needed to identify the magnitude of AMPK phosphorylation following RE and AE with various intensities.

This is the first study comparing the time course of changes in CaMKII phosphorylation between RE and AE. The results showed that CaMKII was phosphorylated immediately after both acute RE and AE. This was consistent with a previous study showing that the phosphorylation of CaMKII was increased significantly immediately after acute AE (Rose et al. 2006). Together, these results demonstrate that both acute RE and AE induce CaMKII phosphorylation immediately after exercise.

The phosphorylation of CaMKII apparently depended on Ca^2+^ released from the sarcoplasmic reticulum. Ca^2+^ release is increased by higher skeletal muscle contraction tension (Chin and Allen 1997). Accordingly, RE, which induces higher contraction tension than AE, might phosphorylate CaMKII to a greater extent than AE. However, the phosphorylation of CaMKII after acute RE returned to the baseline level faster than after acute AE. Thus, contraction tension might not be the most significant regulatory factor for CaMKII phosphorylation after AE.

As a downstream signal of AMPK and CaMKII, the phosphorylation of TBC1D1 (Ser237) was measured after acute RE and AE and found to match the time course of changes in AMPK phosphorylation. To better support the functional relationship between these changes, TBC1D1 phosphorylation was measured in response to AICAR‐induced AMPK phosphorylation or insulin/IGF1 stimulation in vitro. The results showed that AMPK phosphorylation induced TBC1D1 phosphorylation. In contrast, insulin/IGF1‐induced Akt signal activation did not affect TBC1D1 phosphorylation at Ser237. A previous animal study showed that p‐TBC1D1 (Ser237) increased immediately after acute AE, and was diminished in AMPK knockout mice (Fentz et al. 2015). Based on these results, acute RE‐induced TBC1D1 phosphorylation at Ser237 may be also caused by AMPK phosphorylation.

Acute RE‐induced IGF1 expression was assessed previously as a regulator of skeletal muscle hypertrophy (Ogasawara et al. 2013b). As a glucose uptake regulator, IGF1 was measured after acute RE and AE. The results showed that skeletal muscle IGF1 was increased 1 h after acute RE and maintained until 3 h. In contrast, acute AE did not augment intramuscular IGF1. Following augmented IGF1 expression, the increase in Akt phosphorylation seen in the RE was more prolonged than that in the AE group. As a downstream signal of Akt, AS160 phosphorylation was also maintained longer than AE. Moreover, using an in vitro model, we also determined that IGF1 stimulated Akt and AS160 phosphorylation. According to these results, Akt and AS160 phosphorylation after RE may be modulated by RE‐induced IGF1 expression, which may have contributed to prolonged increase in Akt and AS160 phosphorylation. Taken together, the IGF1 signaling, including prolonged Akt and AS160 phosphorylation, may be a specific signal response to acute RE.

The early‐phase phosphorylation of Akt by both RE and AE may occur independently of IGF1 expression because there was no change in IGF1 expression immediately after exercise. Moreover, according to a previous study, the integrin/Akt signaling response occurred immediately after exercise (Klossner et al. 2009). Thus, Akt phosphorylation immediately after acute RE and AE may be caused by an integrin/Akt signal.

This is the first study which directly compared AS160 phosphorylation in response to RE and AE. Previous studies showed that AS160 phosphorylation in rat skeletal muscle after swimming increased immediately after exercise, and it maintained until 3 h after exercise (Arias et al. 2007; Funai et al. 2009; Castorena et al. 2014). This is contrary to this study which demonstrated that AS160 phosphorylation returned to baseline level by 3 h after exercise. However, these previous studies used different mode of exercise (i.e., swimming vs. running) and analyzed epitrochlearis muscle. Acute swimming exercise‐induced PGC1*α* expression, which is downstream of AMPK, was different with acute running exercise on several skeletal muscles including epitrochlearis and gastrocnemius muscle (Terada and Tabata 2004). Additionally, epitrochlearis and gastrocnemius muscle have different muscle fiber composition (Castorena et al. 2011). These methodological differences between previous and this studies may have caused the differences in AS160 phosphorylation level and time course of response.

This is also the first report that compared the time course of changes in GLUT4 translocation and upstream signal responses after acute RE and AE. Interestingly, enhanced GLUT4 translocation after acute RE was found at later time points, but was maintained longer than after acute AE. Previously, acute AE was reported to augment GLUT4 translocation immediately after exercise, and GLUT4 translocation returned to the baseline level by 2 h after exercise (Goodyear et al. 1990). Additional important results were that the RE‐induced increase in AS160 phosphorylation was more prolonged than TBC1D1 phosphorylation, and that acute RE‐induced GLUT4 translocation was also prolonged until the same time point. Furthermore, the induction of AS160/TBC1D1 phosphorylation and GLUT4 translocation was not as prolonged with AE as with RE. These findings may underscore the significance of AS160 signal activation on RE‐induced GLUT4 translocation. In the future study, we need to directly assess the glucose uptake to confirm our findings on GLUT4 translocation.

Overall, our data showed that AMPK/TBC1D1 and IGF1/Akt/AS160 signal activation enhanced glucose uptake independently, and that acute RE activated these signals. Moreover, acute RE increased the expression of skeletal muscle IGF1 as a RE‐specific GLUT4 translocation regulator. Further studies might clarify the contribution of IGF1 signals to acute RE‐induced GLUT4 translocation by using an IGF1 knockout model.

## Conflicts of Interest

The authors declare no conflicts of interest, financial or otherwise.
